# On Complex Formation between 5-Fluorouracil and β-Cyclodextrin in Solution and in the Solid State: IR Markers and Detection of Short-Lived Complexes by Diffusion NMR

**DOI:** 10.3390/molecules25235706

**Published:** 2020-12-03

**Authors:** Daria L. Melnikova, Zilya F. Badrieva, Mikhail A. Kostin, Corina Maller, Monika Stas, Aneta Buczek, Malgorzata A. Broda, Teobald Kupka, Anne-Marie Kelterer, Peter M. Tolstoy, Vladimir D. Skirda

**Affiliations:** 1 Institute of Physics, Kazan Federal University, Kremlevskaya 16a, 420111 Kazan, Russia; melndaria@gmail.com (D.L.M.); badrievaz@gmail.com (Z.F.B.); 2 Institute of Chemistry, St. Petersburg State University, Universitetskiy pr. 26, 198504 St. Petersburg, Russia; kostin-micha@mail.ru; 3 Institute of Physical and Theoretical Chemistry, Graz University of Technology, NAWI Graz, Stremayrgasse 9, 8010 Graz, Austria; maller.c@gmx.at (C.M.); kelterer@tugraz.at (A.-M.K.); 4 Department of Chemistry, Opole University, Oleska Street 48, 45-052 Opole, Poland; mstas@uni.opole.pl (M.S.); abuczek@uni.opole.pl (A.B.); broda@uni.opole.pl (M.A.B.)

**Keywords:** 5-FU, β-CD, inclusion complex, IR, NMR, molecular modeling

## Abstract

In this work, the nuclear magnetic resonance (NMR) and IR spectroscopic markers of the complexation between 5-fluorouracil (5-FU) and β-cyclodextrin (β-CD) in solid state and in aqueous solution are investigated. In the attenuated total reflectance(ATR) spectra of 5-FU/β-CD products obtained by physical mixing, kneading and co-precipitation, we have identified the two most promising marker bands that could be used to detect complex formations: the C=O and C-F stretching bands of 5-FU that experience a blue shift by ca. 8 and 2 cm^−1^ upon complexation. The aqueous solutions were studied by NMR spectroscopy. As routine NMR spectra did not show any signs of complexation, we have analyzed the diffusion attenuation of spin–echo signals and the dependence of the population factor of slowly diffusing components on the diffusion time (diffusion NMR of pulsed-field gradient (PFG) NMR). The analysis has revealed that, at each moment, ~60% of 5-FU molecules form a complex with β-CD and its lifetime is ca. 13.5 ms. It is likely to be an inclusion complex, judging from the independence of the diffusion coefficient of β-CD on complexation. The obtained results could be important for future attempts of finding better methods of targeted anticancer drug delivery.

## 1. Introduction

**Figure 1:**
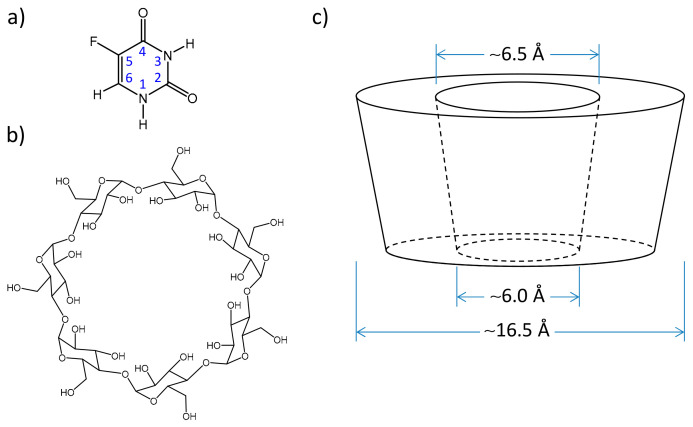
Structure and atom numbering of 5-fluorouracil (5-FU) (**a**), chemical formula of β-cyclodextrin (β-CD) (**b**) and its dimensions (**c**).

One of the most accessible and abundant substances for the chemotherapy of the majority of cancer forms is 5-fluorouracil (5-FU). This chemical acts by binding to the target thymidylate synthase protein, interrupting the synthesis of pyrimidine thymidine, which is required for DNA replication, thus inhibiting cell growth [1,2]. Despite its broad antitumor activity, 5-FU exhibits a number of pharmacological limitations, especially in case of cancer relapse and metastasis, for which many patients show multiple drug resistance [3]. For the pharmacological effectiveness, there is an optimal concentration of the drug in the patient’s blood stream [4,5], which has the required therapeutic effects and at the same time limits the undesired toxic side effects. For 5-FU, the most common side effect is the suppression of hematopoiesis (production of the cellular components of blood) [6,7,8]. Due to the untargeted delivery of 5-FU and its distribution within the body, in order to maintain the required concentration at the given location, large amounts of 5-FU are being administered or it is being administered more frequently [9]. Additionally, the limited bioavailability of 5-FU has been reported previously [10,11,12,13]. Moreover, large variations of the rates of intracellular metabolic reactions leading to the formation of thymidylate synthase, its cofactor 5,10-methylenetetrahydrofolate and other chemicals involved in the process of cell growth inhibition by 5-FU were observed. There are several methods of improving the pharmacological effectiveness of 5-FU [2,14]. For example, 5-FU can be used in combination with drugs such as leucovorin (folinic acid) [15,16], isoflavone genistein [17] and others, influencing the transport of methotrexate through the cell membrane, thus reducing the toxicity of 5-FU. It also has to be mentioned that 5-FU is poorly soluble in water (ca. 1 mg/mL [18]) and its solutions are often prepared in strongly basic media which also increases the side effects of the treatment [19]. The molecule of 5-FU, as a typical pyrimidine base, is subject to tautomeric equilibria between several structural isomers (NH/OH tautomers) [20,21,22]. Despite the possible existence of six tautomers, only one of them (diketo C=O/C=O form, see Figure 1a) is present practically exclusively in aqueous solutions [23] and this isomer is active as a chemotherapeutic agent [24,25]. Thus, the stabilization of the diketo tautomer and its protection against various destructive factors (oxidation, enzymatic decomposition, sorption and evaporation) are also required for the effectiveness of 5-FU.

In summary, the improved bioavailability of 5-FU due to the increase in its solubility and stability in the blood stream and prolonged duration of its antitumor cytotoxicity are the focal problems of effective 5-FU chemotherapy. Many of these issues could be addressed by encapsulation of 5-FU in molecular containers serving as hosts or protective shells. One of the most commonly applied types of such containers, actively used as complexation agents in supramolecular chemistry, are cyclodextrins (CDs) [26,27,28,29]. These host molecules are cyclic oligomers of glycopyranose, obtained by enzymatic treatment of starch [30]. The CDs are well-soluble in water, biocompatible, non-toxic, biodegradable and relatively cheap. Depending on the number of subunits in the macrocycle (six, seven or eight) the CDs are called α-, β- or γ-CD. The structure of β-CD is shown in Figure 1b. The encapsulation of guest molecules usually occurs inside the cavity of CDs (see their slightly conical shape [27] and dimensions [31], shown schematically in Figure 1c for β-CD). Typically, for the formation of a stable complex, the size of the guest molecule should “fit” the size of the cavity. Small molecules usually form 1:1 complexes with CDs [32], though in some cases a complex involving two host molecules per one guest molecule could be formed [33]. The OH groups are located on the outer surface of the CD molecule (precisely, at lower and upper rims of a cage). This makes the inner surface of the cavity more hydrophobic [34] and often increases its affinity towards guest molecules, which are poorly soluble in water. In aqueous solutions, the hydrophobic cavity of β-CD could hold up to 10 water molecules which are displaced upon complexation with less polar guests [35,36]. The guest molecules are held in the cavity by hydrophobic interactions, van der Waals forces and hydrogen bonds [37,38,39], often strongly restricting guests’ rotational degrees of freedom [40]. The formation of host–guest complexes involving CDs is possible not only with non-polar (hydrophobic) compounds, but also with organic acids, amides, several ions and even gases [41,42]. Inclusion complexes could be studied by a number of methods [43]: fluorescence [44], Raman or IR spectroscopy [45,46], liquid state chromatography [47], electron paramagnetic resonance (EPR) [48], nuclear magnetic resonance (NMR) spectroscopy, etc. [49].

Encapsulation of 5-FU in a CD molecule might lead to the formation of a well-soluble, non-toxic complex, increase the period of its circulation in the blood and modulate the rate of its metabolic decomposition, as well as the release rate of 5-FU in tissues. The dimensions of both the host and its guest molecule seem to match well: ca. 6.5 Å for both the β-CD cavity [31] and 5-FU [50], allowing one to expect the formation of a 1:1 complex (see the dimensions of the β-CD molecule in Figure 1c). The formation of 5-FU/β-CD inclusion complexes in aqueous solution was indicated by UV spectroscopy, as well as in solid state (physical mixture and kneaded mixture) by powder X-ray diffraction and FT-IR spectroscopy [51].

Concerning NMR spectroscopy, a wide set of available techniques in principle allows not only to detect the host–guest complex formation, but also to study its structure and dynamics of formation/decomposition. In this regard, the use of a combination of pulsed-field gradient (PFG) NMR methods, including NMR relaxometry and diffusion ordered NMR spectroscopy (DOSY) is promising, as it could provide information about the translational and rotational mobility of molecules participating in the supramolecular assembly (motional dynamics of the guest molecules inside the host cavity, the type of host–guest bonding, etc.), the structure and the stability of the complex [52,53,54].

Several experimental studies have claimed the detection of 5-FU/β-CD inclusion complexes using various approaches [55,56,57]. However, Kavitha et al. [56] reported IR spectra of solid products obtained by physical mixture, kneading, co-evaporation and freeze-drying method and observed no visible changes of 5-FU bands in the presence of β-CD. In another study, 5-FU and β-CD were dissolved in double distilled water and dried [58], but no clear evidence from IR spectra could be noticed of 5-FU/β-CD inclusion complex formation in the solid state. Additionally, calorimetric studies reported by Terekhova et al. [59] did not support the complexation of 5-FU and β-CD in solution. Later on, the same group studied complexation of uracil and lumazine with β-CD in pure water by NMR spectroscopy [57]. The observed changes of ^1^H NMR signals were within the limits of measurement error (below 0.005 ppm). The authors have attributed the apparent lack of evidence for complexation to the different impact of ions in the studied buffers. From the above reports, we conclude that no consensus in favor of or against the 5-FU/β-CD complex formation has been reached yet. Thus, further experimental work is needed in this regard.

To study the process of complexation between 5-FU and β-CD in this work, we applied the following techniques:

(a) NMR spectroscopy (standard 1D and rotating-frame nuclear Overhauser effect spectroscopy (ROESY) NMR, PFG NMR in aqueous solution at room temperature and neutral pH);
(b) IR spectroscopy (ATR for the solid-state mixtures of 5-FU and β-CD prepared by physical mixing, kneading and co-precipitation);
(c) Density Functional Theory (DFT) calculations (gas phase; structure optimization and theoretical IR spectra in the gas phase).

The main questions we address in this work are as follows: (i) Are 5-FU/β-CD complexes formed using mechanochemical synthesis in the solid state and could the IR spectroscopy detect them? (ii) Are these formed in aqueous solution and which NMR parameters are suitable to detect them? In case of complexation, additional questions arise: (iii) How stable they are? (iv) What is their average lifetime in solution? (v) How rigid is the 5-FU molecule encapsulated inside β-CD?

## 2. Results and Discussion

### 2.1. ATR Spectra

**Figure 2:**
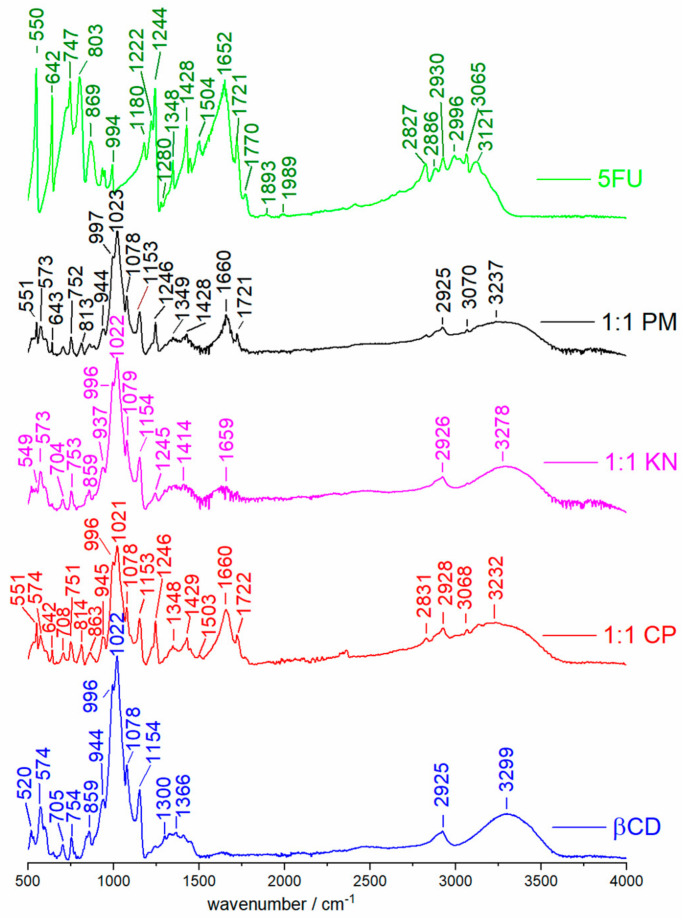
The ATR spectrum of 5-FU shows characteristic absorption bands in the range of 3121–2827 cm^−1^ which are due to the hydrogen-bonded N-H group vibrations and the C-H stretching vibrations. The C=O, C-N and C=C vibrations appear as strong bands in the region of 1770–1280 cm^−1^, with the most intensive carbonyl stretch at 1652 cm^−1^. The C-F stretching band appears at 1244 cm^−1^. The bands in the fingerprint region are due to vibrations of the substituted pyrimidine ring (994–550 cm^−1^). In agreement with the literature [60], the ATR spectrum of β-CD shows a broad and strong O-H band in the region of 3600–3000 cm^−1^ and several narrower vibration bands in the region of 1500–1250 cm^−1^. The absorption band at 2925 cm^−1^ is due to the stretching vibrations of aliphatic CH_2_ groups. The most intensive band at 1022 cm^−1^ is due to the C-O stretching vibration. Bands in the fingerprint region are due to ring vibrations (859–520 cm^−1^).

The vibrational spectra of 5-FU/β-CD mixtures were studied for solid-state systems. Figure 2 presents the ATR spectra of dry neat β-CD, neat 5-FU and the 1:1 products obtained by kneading (KN), physical mixing (PM) and co-precipitation (CP).

In the case of ATR spectra of PM and CP products, it is possible to observe very weak CH and NH stretch bands of the 5-FU guest molecule, overlapping with a strong, broad band due to hydrogen-bonded OH groups of the β-CD host molecule. The characteristic C=O stretching band of 5-FU at 1652 cm^−1^ undergoes a blue shift by 8 cm^−1^ in PM and CP samples while the sharp band at 1721 cm^−1^ remains unchanged. In addition, the C-F stretching band shifts from 1244 cm^−1^ in neat 5-FU to 1246 cm^−1^ in the solid-state products. The apparent shifts, namely, shifts of C=O and C-F stretching bands by 8 and 2 cm^−1^, respectively (Figure 2), suggest a direct intermolecular interaction between 5-FU and β-CD and a potential complex formation. This is also confirmed in the ATR spectrum with increased 5-FU concentration (5-FU:β-CD 5:1, see Supplementary Materials Figure S1). Unfortunately, the broad and unresolved OH and NH stretching vibration bands above 3000 cm^−1^ appeared to be rather non-informative.

Previously, Di Donato et al. [51] claimed a formation of a 5-FU/β-CD complex and presented spectra of free and complexed drugs by the ATR method. They also observed a blue shift of C=O stretch band at about 1656 cm^−1^ and some broadening of other 5-FU peaks in their complexes with α- and β-cyclodextrins. Our current IR study supports their observations and indicates that the most promising diagnostic band for 5-FU/β-CD complexation is the carbonyl stretching band (observed at 1652 cm^−1^ in our ATR spectra), which experiences a moderate blue shift. Additionally, a small blue shift is also observed for the C-F stretch band (see Figure 2). It is interesting to compare these results with some recent IR studies on formation of inclusion complexes between selected drug molecules and β-CD. For example, no clear modification of IR spectra of omeprazole [61] was noticed, whereas in valdecoxib [62] the sulfonamide stretch shifted by 4–7 cm^−1^, similar to the C=O and C-F stretches in our complexes, and in taladafil [63] the C=O band increased its intensity when forming solid-state complexes with β-CD prepared by PM or KM methods.

### 2.2. Quantum Chemical Calculations: Geometry

**Figure 3:**
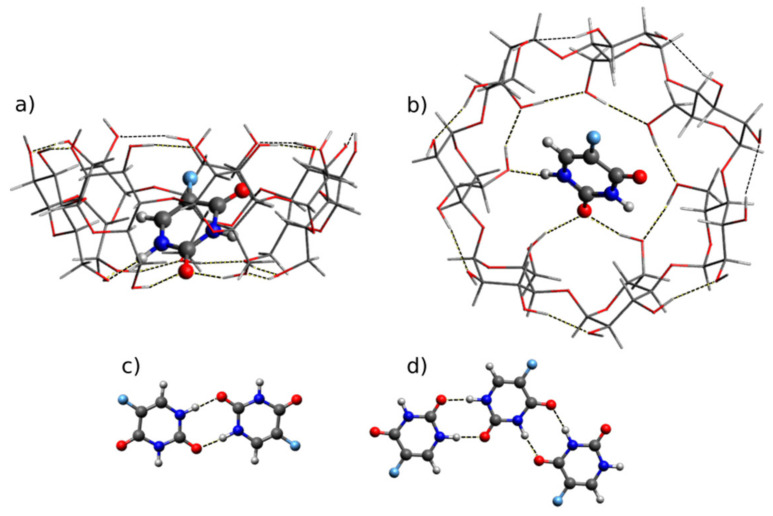
B3LYP-D3/6-31+G(d,p) calculated structures of (**a**,**b**) the most stable complex of 5-FU with β-CD (*E*_int_ = −195 kJ/mol, [37]) and (**c**) the 5-FU dimer and (**d**) trimer.

In our previous theoretical study [37], we have demonstrated that in the most stable complex of 5-FU with β-CD, the diketo tautomer of 5-FU is placed almost vertically inside the β-CD cavity, forming strong NH···O hydrogen bonds with the smaller rim (O···H distances range from 1.6 to 2.0 Å), at the same time destroying the network of intramolecular OH···O hydrogen bond network—see Figure 3a (side view) and 3b (top view). This geometry is consistent with an earlier docking study [51]. Other higher-energy inner and outer complexes are ignored for this work, because they were found to be much less stable by more than 30 kJ/mol. Alternative orientations of OH···O hydrogen bond networks at smaller and larger rims of β-CD were also not considered in this work, because according to ref. [64] they differ by no more than 13 kJ/mol and do not influence the calculated IR spectra significantly.

The structures of the isolated 5-FU diketo dimer, (5-FU)_2_, and of the trimer, (5-FU)_3_, surrounded by a polar environment (modeled by a polarizable continuum), are held by several NH···O=C hydrogen bonds, as shown in Figure 3c,d. Such hydrogen bonding approximately represents intermolecular interactions in the crystal lattice, which consists of infinite hydrogen-bonded chains [65]. In order to predict changes in the vibrational spectra of 5-FU due to complexation with β-CD, both the dimer and the trimer structures of the drug were considered as models of crystalline 5-FU.

### 2.3. Quantum Chemical Calculations: Predicted IR Spectra

**Figure 4:**
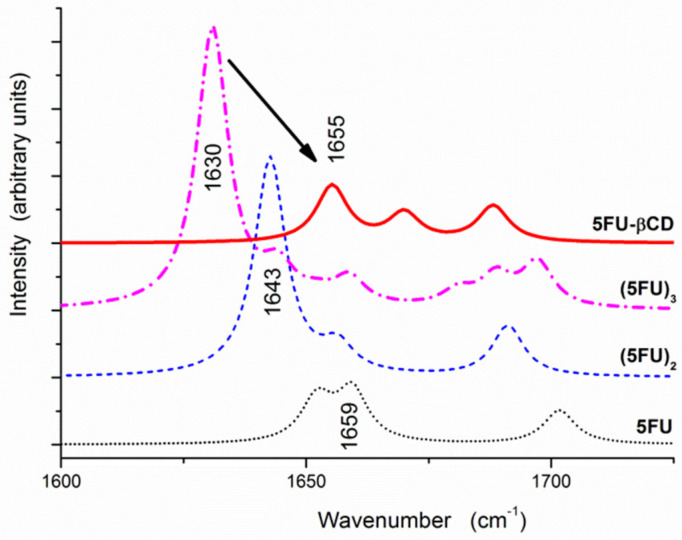
Fragments of theoretical IR spectra containing C=O and C=C stretching vibrations, calculated for the 5-FU monomer, dimer and trimer, as well as the 5-FU/β-CD complex. The calculated harmonic wavenumbers were scaled by a factor of 0.968 [66].

In Figure 4 are gathered the theoretical IR spectra in the C=O/C=C stretching region (1725–1600 cm^−1^) of the 5-FU monomer, the dimer (5-FU)_2_ and the trimer (5-FU)_3_, as well as the 5-FU/β-CD complex, calculated using the geometries described above. A typical red shift of the C=O stretching band due to formation of NH···O=C hydrogen bonds is apparent for (5-FU)_2_ (1643 cm^–1^) and (5-FU)_3_ (1630 cm^−1^), moving from 1652 to 1659 cm^−1^ for the 5-FU monomer. Interestingly, this band appears at 1655 cm^−1^ in the 5-FU/β-CD complex and is ca. 25 cm^−1^ blue-shifted with respect to the corresponding vibration in the trimer. This blue shift is due to the elongation of the hydrogen bond formed by the C=O group in the 5-FU/β-CD complex in comparison to the 5-FU dimer or trimer. The O···H distance is 1.84 Å for C4=O···HO_β-CD_ in the 5-FU/β-CD complex and 1.78 Å for C4=O···HN in (5-FU)_2_, respectively.

Besides, in the theoretical IR spectra the predicted unscaled wavenumbers of C-F stretching vibration of 5-FU for its monomer, dimer, trimer and the 5-FU/β-CD complex are 1245, 1255, 1246–1254 and 1260 cm^−1^, respectively. This blue shift of 4 to 14 cm^−1^ upon complexation with β-CD matches reasonably well the experimentally measured blue shift of 2 cm^−1^ from 1244 cm^−1^ in crystalline 5-FU to 1246 cm^−1^ in its solid products with β-CD (see Figure 2).

In summary, the theoretical IR spectra have qualitatively reproduced the effect of complexation observed experimentally: the disruption of hydrogen bonds in the 5-FU crystal and the formation of somewhat weaker hydrogen bonds in the inclusion complex manifests itself as a moderate blue shift of the C=O band and a small blue shift of the C-F stretching band.

### 2.4. NMR Measurements

**Figure 5:**
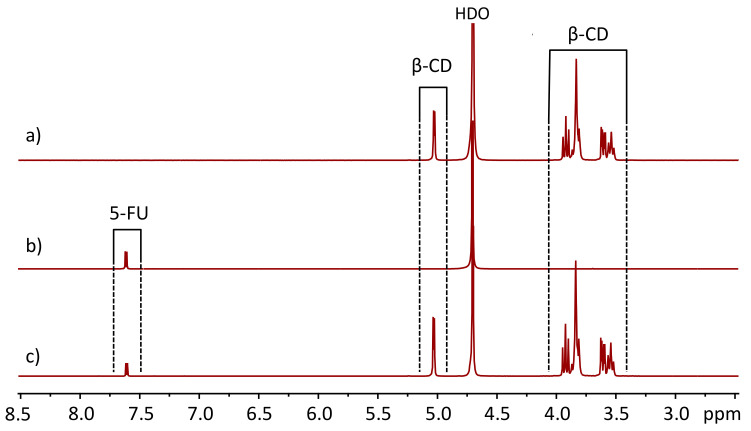
The ^1^H NMR spectra (298 K) of solutions of (**a**) β-CD, (**b**) 5-FU and (**c**) their equimolar mixture in D_2_O.

The NMR studies were carried out using aqueous solutions of 5-FU and β-CD. Thus, in contrast to the solid-state systems described above, here interactions with the surrounding water molecules play a crucial role in determining the properties of complexes, such as their stability, lifetimes, dynamics of translational diffusion and conformational reorganization. Figure 5 shows an overview of ^1^H NMR spectra of solutions of β-CD, 5-FU and their equimolar mixture in D_2_O. The anomeric proton of β-CD resonates at ca. 5 ppm, while the other protons give rise to a set of signals at 3.5–4.0 ppm (Figure 5a). The CH proton of 5-FU resonates at ca. 7.6 ppm and it is split into a doublet due to the spin–spin coupling constant ^3^*J*_HF_ = 5.3 Hz (Figure 5b). Both NH protons of 5-FU are chemically exchanged with the solvent and are not visible. The chemical shifts and coupling constants remain virtually unchanged upon mixing the equimolar amounts of β-CD and 5-FU (Figure 5c, Figure S2). In the first approximation, this might be interpreted as an indication of the absence of interaction between the host and guest molecules. To a certain extent, this interpretation would also be consistent with some of the previously published data [67], which showed that for the investigation of complexation between β-CD and several small molecules the traditionally used NMR methods might be non-informative, as the changes in NMR observables upon complexation are too small. In our experiments, the changes in chemical shifts, coupling constants and T_1_ relaxation times were too small to be conclusive (Figures S2 and S3). Additionally, we were unable to detect any cross peaks between β-CD and 5-FU in ROESY spectra [68] (see Figure S4).

**Figure 6:**
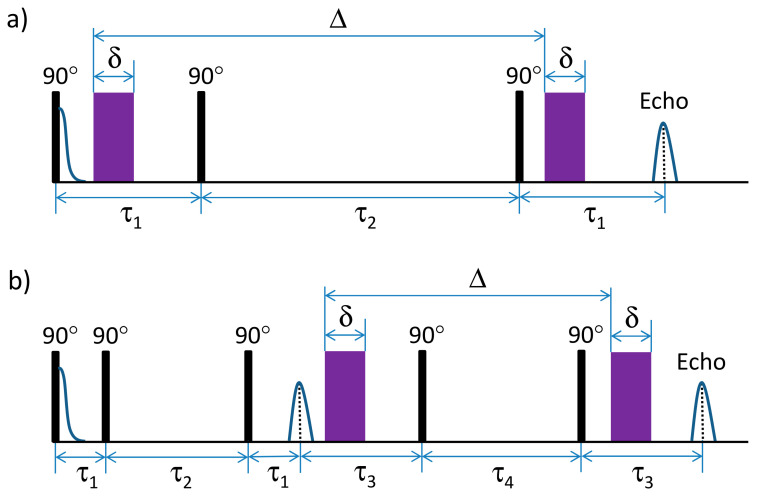
The pulse sequences used in this work for the detection of the diffusion attenuations of spin–echo signals: (**a**) standard pulse sequence and (**b**) modified 5-pulse sequence. The pulsed-field gradients (PFGs) are shown in purple.

In order to better characterize the studied molecular system in solution, self-diffusion coefficients (hereinafter referred to simply as diffusion coefficients) were measured using the stimulated-echo pulse sequence, shown schematically in Figure 6a (PGSTE) [69] and modified five-pulse stimulated echo sequence [70] (see Figure 6b). The purpose and benefits of the modified pulse sequence will be described below.

The translational diffusion of ^1^H nuclei was studied by varying the PFG amplitude, *g*, while keeping all other parameters of the pulse sequence fixed. To establish the influence of spin–spin and spin–lattice relaxation times, several experiments were performed at various time intervals with $\tau_{1}$ varying between 2 and 4 ms, while the diffusion time $t_{d\;}=\;\Delta -\delta /3$ varied (by changing $\tau_{2}$) between 7 and 30 ms. In all cases, the PFG duration was kept at 0.62 ms.

For multicomponent systems, the diffusion attenuation could be described by the following multiexponential function [71,72]:

(1) $$\frac{A\left(g^{2}\right)}{A\left(0\right)}=\underset{i}{\sum }p_{i}^{\prime }\cdot exp\left(-\gamma^{2}g^{2}\delta^{2}D_{i}t_{d}\right)$$

where the sum goes over all the components of the system. $A\left(g^{2}\right)$ is the overall diffusion attenuation of the spin–echo amplitude, $A\left(0\right)$ is the spin–echo amplitude at *g* = 0, *γ* is the gyromagnetic ratio for protons, *δ* is the PFG duration, $D_{i}$ and $p_{i}^{\prime }$ are diffusion coefficient and observed population fraction of the *i*th component of the studied system measured for the given values of time intervals $\tau_{1}$ and $\tau_{2}$.

In the absence of chemical exchange between the components of the system characterized by different diffusion coefficients, the observed population fractions $p_{i}^{\prime }$ are related to the true population fractions $p_{i}$, as follows:

(2) $$p_{i}^{\prime }=\frac{p_{i}exp\left(-\frac{2\tau_{1}}{T_{2i}}-\frac{\tau_{2}}{T_{1i}}\right)}{\sum_{i}p_{i}exp\left(-\frac{2\tau_{1}}{T_{2i}}-\frac{\tau_{2}}{T_{1i}}\right)}$$

where $T_{1i}$ and $T_{2i}$ are the spin–lattice and spin–spin relaxation times of the *i*th component, respectively.

Despite the fact that the average diffusion coefficient 〈D〉

(3) $$\langle D\rangle =\underset{i}{\sum }p_{i}^{\prime }\cdot D_{i}$$

depends on the temporal parameters of the experiment, it is important to note that the individual diffusion coefficients $D_{i}$ are measured correctly for the given values of $\tau_{1}$ and $\tau_{2}$. Population fraction $p_{i}$ could be estimated by performing several measurements and extrapolating the $p_{i}^{\prime }$ value to the zero durations of $\tau_{1}$ and $\tau_{2}$.

When the chemical exchange is present, Equation (2) is not fulfilled and Equation (1) is further complicated by the effective distribution of diffusion coefficients with values intermediate between $D_{i}$ values. Thus, the correct information about the chemical exchange dynamics could be obtained only if in the analysis of the diffusion attenuation curves the factors related to the distribution of T_1_ and T_2_ times will be excluded. This is possible by using the modified pulse-sequence schematically shown in Figure 6b. By keeping $\tau_{2}+\tau_{4}=const,$ the diffusion attenuation could be detected for various diffusion times $t_{d}$ with constant contribution from the spin–lattice relaxation T_1_. Despite the fact that the second stimulated echo signal is only half as intensive as the first one, the modified pulse sequence allows one to characterize the exchange process between the components with different diffusion coefficients [70], even if there is a distribution of relaxation times within each of the exchanging components. The contribution from the spin–lattice relaxation T_1_ could be determined by comparing the results of the standard (Figure 6a) and modified (Figure 6b) pulse sequences. Previously, the five-pulse sequence was used to measure lifetimes of OH protons in sucrose in aqueous solutions, which was estimated to be as short as ca. 1.5 ms [73].

**Figure 7:**
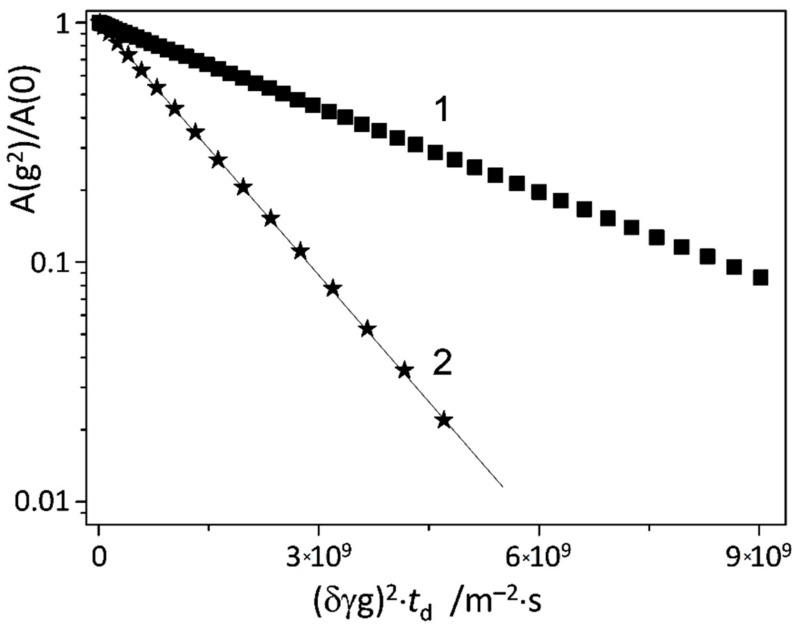
Diffusion attenuations of spin–echo signals for the anomeric CH proton of β-CD (1, squares) and CH proton of 5-FU (2, stars). The sample contained only β-CD (1) or only 5-FU (2) in D_2_O solution at 298 K.

The apparent insensitivity of NMR parameters of 5-FU to the presence of β-CD could be either an indication of the small spectral changes upon complexation or an indication that the lifetime of complexes is too short and the equilibrium is shifted towards the monomers. This dilemma calls for a different set of NMR techniques to characterize the complexation process. We have applied pulsed-field gradient NMR (PFG NMR, “diffusion NMR”), using both standard [69] and modified stimulated echo pulse sequences [70,74,75]. Judging from the spectra shown in Figure 5, the translational diffusion of β-CD and 5-FU individually (not in a mixture) might be estimated, using the diffusion attenuation for the signals in the spectral ranges 7.5–7.8 ppm and 3.5–4.0 ppm, respectively. The resulting plots obtained for the abovementioned spectral regions using the standard stimulated echo pulse sequence are shown in Figure 7. For both β-CD and 5-FU, the diffusion attenuation is a monoexponential decay within the dynamic range of intensities of almost two decimal orders of magnitude. The estimated diffusion coefficients of β-CD and 5-FU differ significantly, as one would expect from the differences in sizes of the molecules: *D*_β-CD_ = (2.700 ± 0.005) 10^−10^ m^2^/s and *D*_5-FU_ = (8.10 ± 0.01) 10^−10^ m^2^/s.

**Figure 8:**
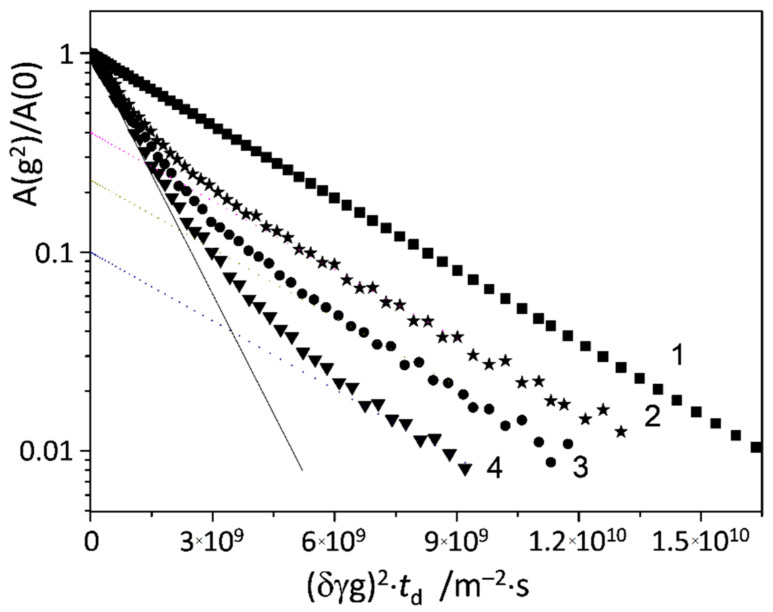
Diffusion attenuations of spin–echo signals for the anomeric CH proton of β-CD (1) and H6 proton of 5-FU (2, 3, 4). The diffusion time $t_{d}$ was 7 ms (curves 1 and 2), 15 ms (curve 3) and 25 ms (curve 4). Dashed lines correspond to Equation (4). Solid line corresponds to the initial slope of curves 2, 3 and 4, which determine the average diffusion coefficient value for 5-FU. The sample contained equimolar amounts of β-CD and 5-FU (0.014 M each) in D_2_O solution at 298 K.

Figure 8 shows the diffusion attenuations obtained for the sample containing both β-CD and 5-FU in equimolar mixture (0.014 M each) and for the same spectral regions using the standard stimulated echo pulse sequence. The diffusion attenuation for β-CD remains monoexponential and could be described by a single diffusion coefficient *D*_β-CD_ = (2.680 ± 0.005) 10^−10^ m^2^/s. In contrast, the diffusion attenuation for 5-FU (curves 2, 3 and 4 in Figure 8) is polyexponential and depends on the diffusion time $t_{d}$. This means that for 5-FU molecules, there is a distribution of diffusion coefficients, which can be described by Equation (1). Note that the final slope of the curves does not depend on $t_{d}$, as all dotted lines in Figure 8 were calculated according to Equation (4)

(4) $$\frac{A\left(G^{2}\right)}{A\left(0\right)}=p_{min}\left(t_{d}\right)\cdot exp\left(-\gamma^{2}g^{2}\delta^{2}D_{min}t_{d}\right)$$

with the same value of *D*_min_ = 2.65 10^−10^ m^2^/s. The observed fraction $p_{min}\left(t_{d}\right)$ of 5-FU molecules having this value of diffusion coefficient decreases with the diffusion time $t_{d}$ (the trend is evident from the faster diffusion attenuation at higher $t_{d}$—see Figure 8; the numerical values will be analyzed below). The initial slopes of the diffusion attenuation curves are equal and could be described by the same average diffusion coefficient value 〈D〉 = (9.10 ± 0.06) 10^−10^ m^2^/s, which does not depends on $t_{d}$.

Such dependence of diffusion attenuation on the diffusion time points towards the presence of a chemical exchange [70,76,77]. Additional experiments conducted using the modified five-pulse sequence—in which the contribution of the spin–lattice relaxation to the diffusion attenuation curves could be excluded—have confirmed this interpretation. In the main text of this work we show only the results obtained using the standard pulse sequence, because the intensity of the second echo is twice lower than that of the first echo, which worsens the signal-to-noise ratio. Thus, the obtained results give evidence for the chemical exchange of 5-FU molecules between at least two states with different diffusion coefficients. As it was mentioned above, one of these states is characterized by the diffusion coefficient *D*_min_ = (2.65 ± 0.05) 10^−10^ m^2^/s. The diffusion coefficient for the second state can easily be calculated using Equation (3) and the assumption of the two-state exchange with the average diffusion coefficient 〈D〉 = (9.10 ± 0.06) 10^−10^ m^2^/s, which gives for the second state *D*_max_ = (9.80 ± 0.08) 10^−10^ m^2^/s. We assume that this state corresponds to free 5-FU molecules because the diffusion coefficient value is close to that observed for the aqueous solution of 5-FU alone, *D*_5-FU_ = (8.10 ± 0.01) 10^−10^ m^2^/s (see Figure 7). In turn, within the margin of error, the *D*_min_ value coincides with that for β-CD molecules, *D*_β-CD_ = (2.680 ± 0.005) 10^−10^ m^2^/s. This is a strong indication that at each moment of time some of 5-FU molecules are in complex with β-CD, sharing the same translational diffusion characteristics. At the same time, there are no measurable differences between diffusion coefficients of free β-CDs and β-CDs in complex with 5-FU. This indicates that the hydrodynamic radii of these species practically coincide, which is consistent with the formation of an inclusion complex (inner host–guest complex), in which the 5-FU molecule is located inside the hydrophobic cavity of β-CD.

**Figure 9:**
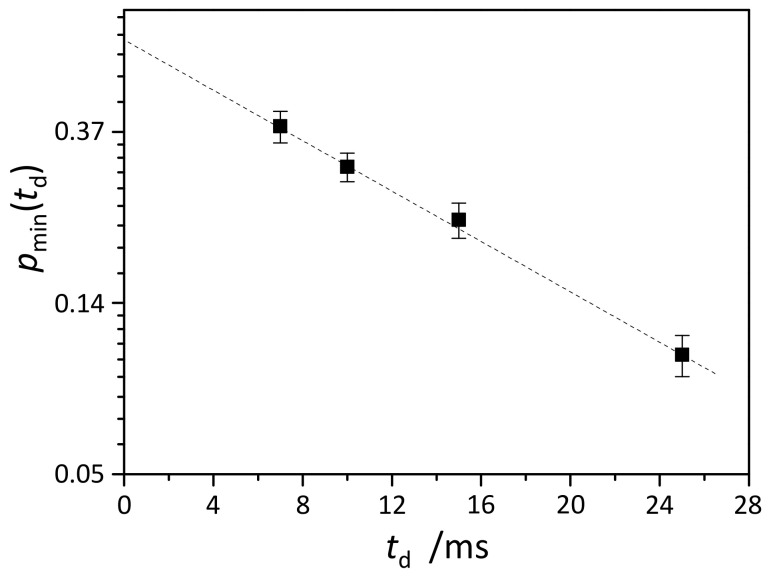
The dependence of the population factor $p_{min}$ on the diffusion time $t_{d}$ obtained from the analysis of the diffusion attenuations measured for 5-FU molecules in equimolar mixture with β-CD in aqueous solution at 298 K. The dotted line was fitted using Equation (6).

Let us now analyze the dependence of diffusion attenuation for 5-FU on $t_{d}$ in more detail. As it was shown in [70], even in the case of a chemical exchange between two states (*a* and *b*) with diffusion coefficients *D_a_* and *D_b_*, the diffusion attenuation could be characterized by a continuous set of intermediate diffusion coefficients. One of the main results of ref. [70] is as follows: if the average diffusion coefficient 〈*D*〉 is independent of $t_{d}$, then the population fractions $p_{i}\left(t_{d}\right)$ (*i* = *a*,*b*) of the limiting states having the defined diffusion coefficients *D_a_* and *D_b_* could be described by a decaying function

(5) $$\frac{p_{i}\left(t_{d}\right)}{p_{i}\left(0\right)}=1-\int_{0}^{t_{d}}\Psi_{i}\left(\tau \right)d\tau$$

where $p_{i}\left(0\right)=\underset{t_{d}\to 0}{\lim}p_{i}\left(t_{d}\right)$ are true population fractions of states *i* = *a*,*b* and the integral represents the probability of the system to leave the corresponding state at least once, accumulated over the duration $t_{d}$. The function $\Psi_{i}\left(\tau \right)$ is the density distribution function of the lifetimes τ in the corresponding states. In other words, the analysis of $p_{i}\left(t_{d}\right)$ (*i* = *a*,*b*) dependencies allows one to determine the function $\Psi_{i}\left(\tau \right)$. For Markovian processes, for which the probability to change states is time-independent, Equation (5) takes a simple exponential form

(6) $$\frac{p_{i}\left(t_{d}\right)}{p_{i}\left(0\right)}=\exp\left(-\frac{t_{d}}{{\bar{\tau }}_{i}}\right)$$

where ${\bar{\tau }}_{i}$ is the average lifetime of the molecule in state *i* = *a*,*b* with the diffusion coefficients *D_a_* or *D_b_*. Figure 9 shows the analysis of the population factor $p_{min}\left(t_{d}\right)$ for the state with the smallest diffusion coefficient *D*_min_. Fitting of the data points using Equation (6) gives

(7) $$p_{min}\left(t_{d}\right)=\left(0.62\pm 0.05\right)\cdot \exp\left(-t_{d}/\bar{\tau }\right),$$

where $\bar{\tau }=13.5\pm 1$ ms. This means that for an equimolar mixture of host and guest molecules (0.014 M each), at any given moment of time, ca. 60% of 5-FU molecules are in complex with β-CD molecules and ca. 40% are free. The distribution function of a complex’s lifetimes is an exponential function with the characteristic lifetime of about 13.5 ms.

Knowing the fraction of 5-FU molecules in complex with β-CD, we could estimate the binding constant K of the reaction 5-FU + β-CD ⇔ 5-FU × β-CD. As the concentrations of both molecules were equal to 0.014 M, we obtain K = 270 M^−1^ (see [67]). This value lies between the values reported in ref. [51] for the 5-FU/β-CD complex measured at pH 7 and 10. Considering that the pH values were not measured in our work, we could say that our results and the results of ref. [51] corroborate each other.

## 3. Experimental Methods

### 3.1. Sample Preparation for Solution-State (High-Resolution) NMR Experiments

The chemicals β-cyclodextrin (β-CD, 1134 g/mol), 5-fluorouracil (5-FU, 130 g/mol) and D_2_O (99.9% D) were purchased from Sigma-Aldrich (Darmstadt, Germany) and used without further purification. The samples were prepared by mixing the calculated volumes of stock solutions of β-CD and 5-FU in the NMR sample tube. The stock solutions were prepared by weighing the chemicals and adding the calculated amounts of D_2_O to the flasks. The resulting concentrations β-CD and 5-FU are given where appropriate.

### 3.2. Sample Preparation for Solid-State IR Experiments

The chemicals 5-FU (>99.0%, Tokyo Chemical Industry (TCI), Tokyo, Japan), β-CD (≥99%, Sigma-Aldrich, Steinheim, Switzerland) (both kept in the dark) and deionized water (99.96%, Euriso-top, Saclay, France) were used without further purification. The solid complexes were prepared according to the literature [60,62,63,78] by using three different methods as described below. After preparation, all samples were dried in an oven at 40 °C for 72 h under vacuum to remove the traces of solvent. The samples were kept in the desiccator for further analyses.

### 3.3. Physical Mixture (PM)

Dried powders of 5-FU and β-CD in a ratio of 1:1 (1 mM each) were mixed and blended in a mortar for 5 min at room temperature.

### 3.4. Kneading Method (KN)

The KN procedure started with dry subtracts, but before mixing a few drops of solvent were added. In this procedure, crystal water may play a role, but one does not expect large amounts of solvent to enter the host cavity, because the material is dried before and after formation of the complexes. The 5-FU/β-CD sample was prepared at room temperature in a 1:1 ratio (1 mM each). Prior to mixing, 3 mL of deionized water was added to β-CD, and 5-FU was dissolved in 3 mL of methanol. A paste was obtained by mixing the two compounds and kneaded manually for 10 min.

### 3.5. Co-precipitation Method (CP)

The CP method started with mixing solutions of the subtracts with subsequent dry-freezing. This might produce a complex with encapsulated solvent molecules. Thus, the PM and KN powders are expected as dry complexes, whereas the CP powder may contain a few molecules of solvent inside the host cavity. The 5-FU/β-CD samples were prepared at room temperature in two ratios, 1:1 (0.15 mM each) and 5:1 (0.15 mM:0.03 mM). 5-FU was dissolved in methanol (20 mL) and sonicated for 30 min. β-CD was dissolved in distilled water (20 mL), stirred with a magnetic stirrer and heated to 50 °C to get a clear solution. Then the 5-FU solution was added dropwise into the β-CD solution and constantly stirred for 5 days at room temperature and was protected from light. After the time, the methanol was evaporated and water was removed using a lyophilisation process (freeze-drying), leaving a white solid.

### 3.6. Attenuated Total Reflectance (ATR) Analysis

ATR spectra were recorded in the 400–4000 cm^–1^ range with resolution of 1 cm^–1^ for the dried solid samples using a Bruker ALPHA FT-IR spectrometer with a Platinum ATR module (Bruker Optik GmbH, Ettlingen, Germany) in single reflection mode, acquiring 128 scans for each sample. The samples were dried at 80 °C before the ATR measurements, in order to remove potential traces of water, which could either be present in purchased β-CD or absorbed due to its hygroscopicity. The results for 1:1 compositions are given in the main text; the results for the CP 5:1 composition are given in the Supporting Information (Figure S1).

As suggested by an anonymous reviewer, it is important to comment on the accuracy of IR peak position determination in our study. Measurement of the FT-IR spectra is easier but the quality is markedly lower than obtained from KBr tablets. Typically, an ATR experiment also introduces some distortions of peak shapes (see, for example, ATR spectra in ref. [51]). However, by using a digital resolution of 1 cm^−1^ in our measurements we could expect an even better estimation of peak position due to curve fitting procedures, used in the spectrometer data processing software. Thus, one could safely estimate the error of peak position determination as ±0.5 cm^−1^. On the other hand, it is important to mention that most peaks of 5-FU measured alone and in the presence of the host molecule were not shifted and observed at the same positions. Thus, one could safely assume a selective shift of two peaks only (stretch C-F and C=O of 5-FU).

### 3.7. Computational Methods

Density Functional Theory (DFT) was applied using the B3LYP functional [79] with inclusion of Grimme’s -GD3 correction for dispersion interaction [80]. The 6-31+G(d,p) basis set was used for geometry optimizations and frequency calculations using the Gaussian 09 program [81]. The choice of the method was validated by comparison of dissociation energy for the water dimer with experimental data (compare ref. [82]). Geometries of 5-FU, 5-FU dimer and trimer, β-CD and the 5-FU/β-CD complex were optimized using tight convergence criterion from the starting geometries as described in ref. [65] for the 5-FU trimer, in ref. [64] for β-CD and in ref. [37] for all other cases. To better model a polar solid-state environment, all structures were optimized using a polarized continuum model (PCM, solvent = water).

It is obvious that a polar environment and hydrogen bonding should somehow be incorporated in our theoretical modeling of structures and vibrational frequencies (one should model the effect of environment). Therefore, it is necessary to take into account the presence of chains of 5-FU molecules held by strong H-bonds in the solid state. There are also strong crystal packing forces present in crystals, partly related to the highly polar environment. Thus, modeling IR spectra of a single (and highly polar) molecule in the gas phase could be an oversimplification. Therefore, models including three molecules instead of two or a single one should better reproduce the experiment and relative shift of characteristic vibrations. We believe that the selected theoretical model is more appropriate than just calculating single molecules in the gas phase.

Harmonic frequencies were calculated using the same level of theory, giving positive frequencies and thus confirming the obtained geometries as minima on the potential energy surface. In the current study, the theoretical IR spectra of the most stable 5-FU/β-CD complex (i.e., the diketo form of 5-FU encapsulated in β-CD, *E*_int_ = −195.5 kJ/mol, K-in-v complex from [37]), the 5-FU dimer and the 5-FU trimer were compared with the experiment (i.e., the ATR results for the solid-state samples).

### 3.8. 1D and 2D NMR experiments

The routine NMR measurements were performed at 298 K using Bruker Avance III 500 NMR spectrometer (11.7 T; 500.13 MHz for ^1^H) at the Center for Magnetic Resonance, St. Petersburg State University. The relevant experimental parameters are given in Figures S1 and S2 in the Supporting Information. Unless specified differently, ^1^H NMR spectra were referenced according to IUPAC recommended procedure [83].

### 3.9. Diffusion NMR

For the diffusion NMR measurements, the equimolar amounts of β-CD and 5-FU were used (0.014 M). All NMR measurements were performed at 298 K using a Bruker Avance III 400 spectrometer (9.4 T; resonance frequency of 400.13 MHz for ^1^H) equipped with a gradient system that allows for a maximum gradient, *g*, of 28 T/m.

## 4. Conclusions

ATR measurements of 5-FU/β-CD products obtained by physical mixing, kneading and co-precipitation in combination with quantum chemical calculations confirm the possibility of formation of the 5-FU/β-CD inclusion complex from separate compounds in the solid state. The presumed structure of this complex involved intermolecular hydrogen bonds between the C=O group of the diketo tautomer of 5-FU and the hydroxyl groups of β-CD smaller rim. The most indicative spectral manifestation of complexation is the blue shift of the C=O stretching band in both the experimental ATR (8 cm^−1^) and the theoretically modeled vibrational spectra (ca. 25 cm^−1^). In addition, the C-F stretch vibration of the drug molecule experiences a small blue shift upon complex formation with β-CD (2 cm^−1^ and 4–14 cm^−1^, for experimental and theoretical IR spectra, respectively).

Significant insights into the dynamics of complexation were obtained by PFG NMR techniques. We have found strong evidence of the formation of the host–guest complex between β-CD and 5-FU in aqueous solution, which is likely to be an inclusion complex, judging from the independence of the diffusion coefficient of β-CD on complexation. However, this complex is not stable, having short lifetimes of ca. 13.5 ms. Unfortunately, this does not qualify β-CD as a good host for a 5-FU drug delivery system. We have shown that routine 1D and 2D NMR experiments are not sensitive enough for the detection and characterization of the β-CD/5-FU complex. The reason is likely to be the short lifetime of the species, as well as the mobility of 5-FU within the complex (as T_1_ and T_2_ relaxation times are not sensitive to complexation).

## Supplementary Materials

The following are available online, Figure S1: ATR spectra of neat 5-FU, the 1:1 CP and the 5:1 CP complexes, and neat β-CD (from top to bottom). Figure S2: The apparent lack of changes of 1H NMR chemical shifts and spin-spin coupling constants upon addition of β -CD to the sample containing solution of 5-FU in D2O. (a) Overview of signal positions anFigure intensities. (b) Fitting of the H6 doublet (values of obtained chemical shifts and coupling constants are added to the figure). The sample preparation conditions are given above. Figure S3. T1 relaxation times (298 K) of H6 proton of 5-FU and selected β-CD signals. Figure S4. ROESY spectrum of 1:1 mixture of 5-FU with β-CD at 298 K. The spectral fragment where cross peaks should appear, in case of complex formation, is marked by a dashed rectangle.
