# Supplementary material for: On Complex Formation between 5-Fluorouracil and β-Cyclodextrin in Solution and in the Solid State: IR Markers and Detection of Short-Lived Complexes by Diffusion NMR
Source: Molecules. 2020 Dec 3;25(23):5706. doi: 10.3390/molecules25235706 (PMC7731325; doi:10.3390/molecules25235706)
Supplement: Supplementary file 1 [file molecules-25-05706-s001.pdf]

*Supporting information for*

# On Complex Formation between 5-Fluorouracil and β-Cyclodextrin in Solution and in the Solid State: IR Markers and Detection of Short-Lived Complexes by Diffusion NMR

**Daria L. Melnikova [1], Zilya F. Badrieva [1], Mikhail A. Kostin [2], Corina Maller [3], Monika Stas [4], Aneta Buczek [4], Malgorzata A. Broda [4], Teobald Kupka [4,*], Anne-Marie Kelterer [3], Peter M. Tolstoy [2,*] and Vladimir D. Skirda [1,*]**

[1] Institute of Physics, Kazan Federal University, Kremlevskaya 16a, 420111 Kazan, Russia; melndaria@gmail.com (D.L.M.); badrievaz@gmail.com (Z.F.B.)

[2] Institute of Chemistry, St. Petersburg State University, Universitetskiy pr. 26, 198504 St. Petersburg, Russia; kostin-micha@mail.ru

[3] Institute of Physical and Theoretical Chemistry, Graz University of Technology, NAWI Graz, Stremayrgasse 9, 8010 Graz, Austria; maller.c@gmx.at (C.M.); kelterer@tugraz.at (A.-M.K.)

[4] Department of Chemistry, Opole University, Oleska Street 48, 45-052 Opole, Poland; mstas@uni.opole.pl (M.S.); abuczek@uni.opole.pl (A.B.); broda@uni.opole.pl (M.A.B.)

* Correspondence: teobald@uni.opole.pl (T.K.); peter.tolstoy@spbu.ru (P.M.T.); kazanvs@mail.ru (V.D.S.)

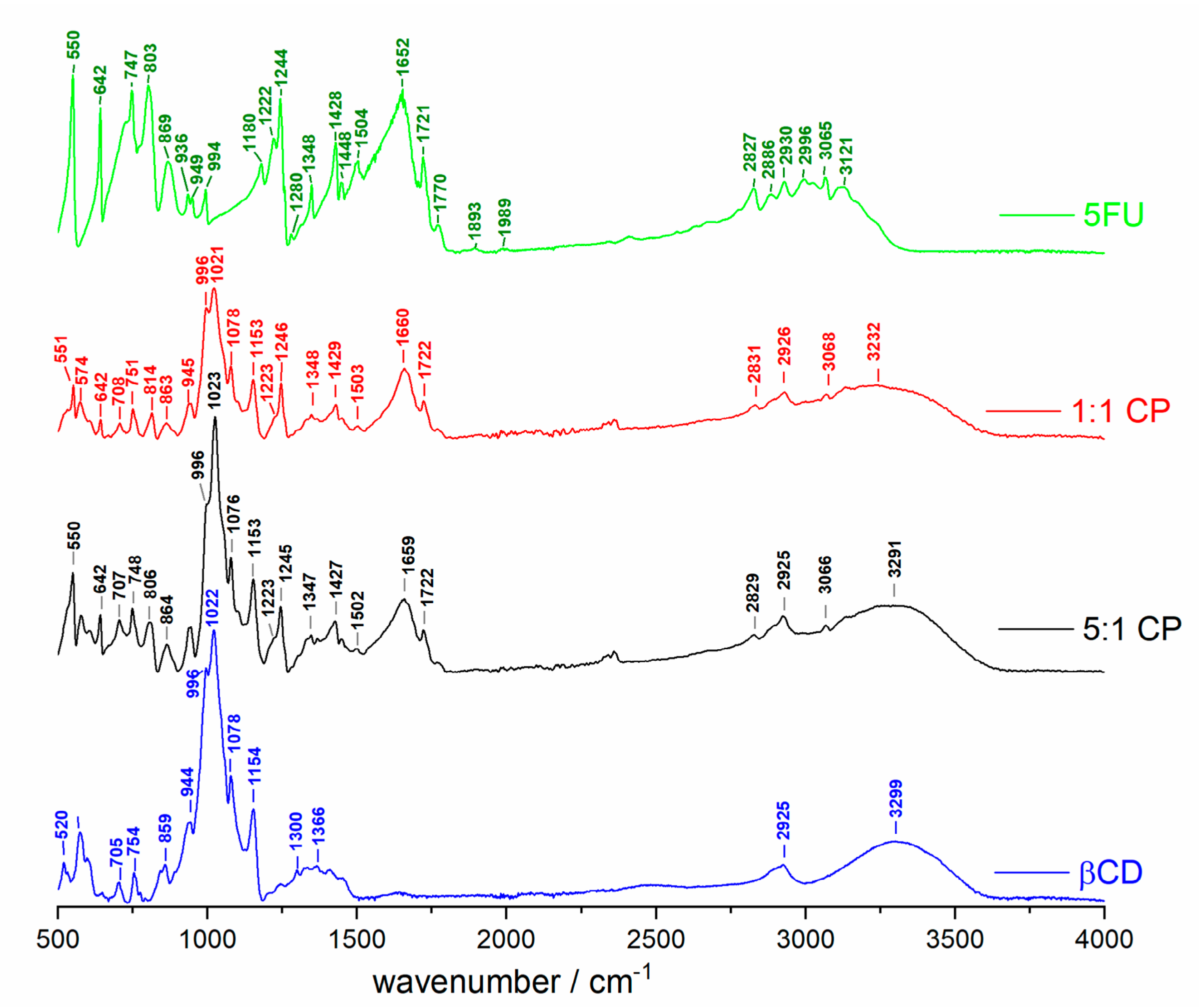


**Figure S1.** ATR spectra of neat 5-FU, the 1:1 CP and the 5:1 CP complexes, and neat β-CD (from top to bottom).

The peak of the 1:1 and 5:1 complexes at 1722 $cm^{-1}$ was scaled to the same intensity to better identify the modification of the peaks when the ratio of 5FU: β-CD is increased from 1:1 (second spectrum from top) to 5:1 (third spectrum from top). The C=O vibration of free 5-FU appears at 1652 $cm^{-1}$ and is blue shifted to 1660 $cm^{-1}$ in the solid-state complexes. The broad band at 3000–3600 $cm^{-1}$ is due to H-bonded hydroxyl groups of β-CD. The C-F stretch band at 1244 $cm^{-1}$ is also blue-shifted in the studied complexes and appears at 1246 $cm^{-1}$.

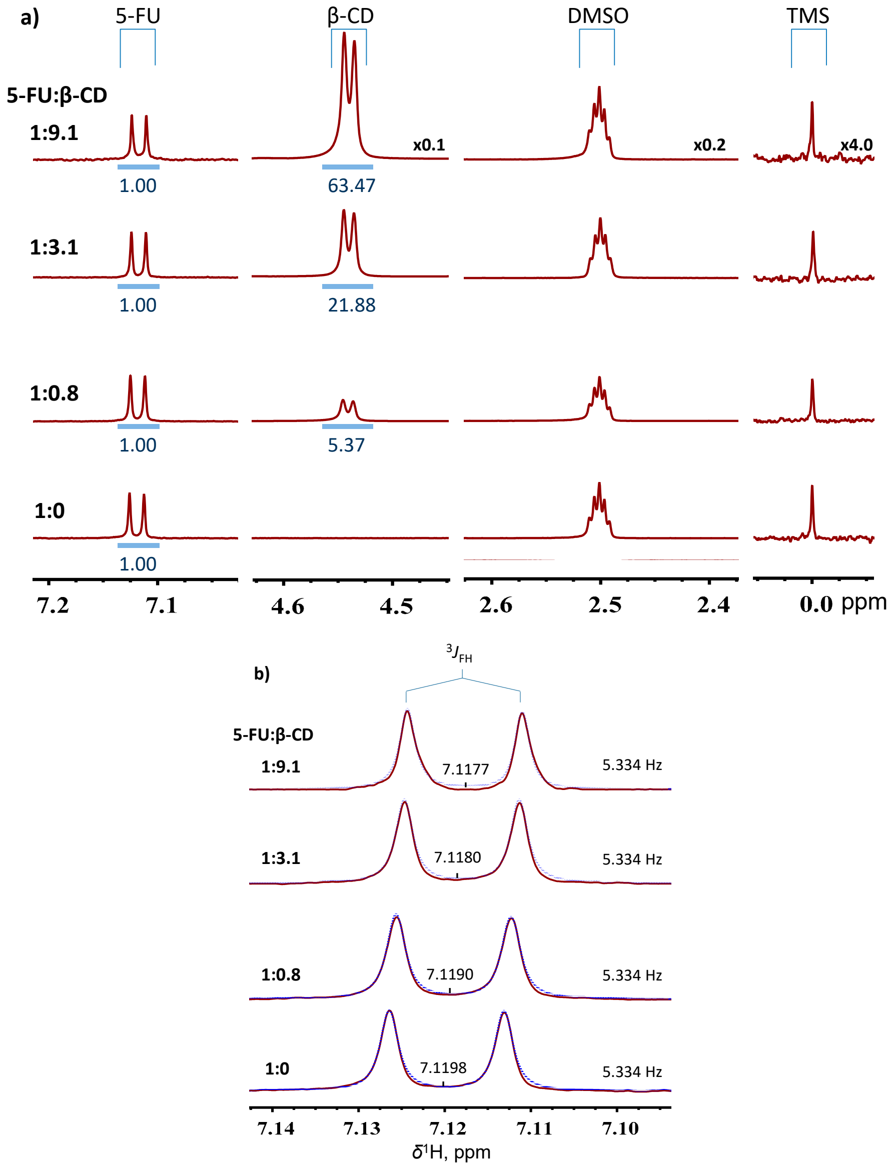


**Figure S2.** The apparent lack of changes of $^1$H NMR chemical shifts and spin-spin coupling constants upon addition of β-CD to the sample containing solution of 5-FU in $D_2O$. (a) Overview of signal positions and intensities. (b) Fitting of the H6 doublet (values of obtained chemical shifts and coupling constants are added to the figure). The sample preparation conditions are given above.

The volume of solution in each sample is 0.6 ml. Absolute concentration of 5-FU was 2.3 mM (0.18 mg) for all samples. Absolute concentrations of β-CD were (from top to bottom) 9.7 mM (6.62 mg), 4.8 mM (3.31 mg), 2.5 mM (1.66 mg) and zero. A capillary containing deuterated dimethylsulphoxide (DMSO-$d_6$) with a small amount of tetramethylsilane (TMS) was placed inside each sample tube. The spectra were referenced to the signal of the residual protons of DMSO (2.501 ppm) as an external standard. Please note that on the one hand this procedure sets the chemical shift of HDO protons at about 4.2 ppm and thus gives a general 0.5 ppm shift of all spectral lines as compared to the spectrum shown in Figure 5 in the main text. On the other hand, referencing to an external standard minimizes the calibration artefacts due to the shift of the HDO/$D_2O$ solvent peak upon addition of the OH-containing β-CD to the sample. In other words, referencing to a signal of the solvent inside a capillary is better suited to track the spectral changes in such a “titration” experiment.

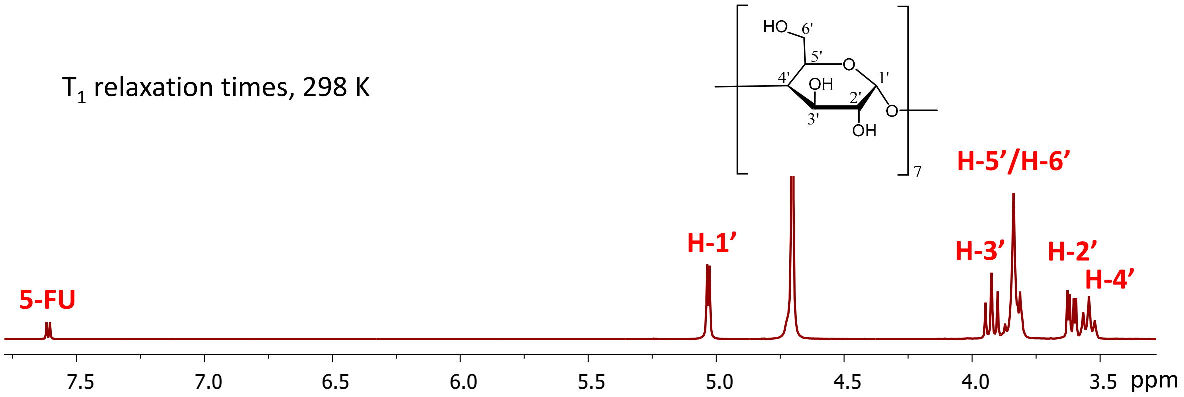


| bCD:5-FU | 5-FU | H-1' | H-3' | H-5'/H-6' | H-2' | H-4' |
|---|---|---|---|---|---|---|
| 1: - | | 0.97 | 1.65 | 0.61 | 1.53 | 0.93 |
| 1:0.5 | 11.2 | 0.97 | 1.67 | 0.61 | 1.55 | 0.94 |
| 1:1 | 11.6 | 0.98 | 1.68 | 0.61 | 1.54 | 0.93 |
| 1:2 | 11.3 | 0.98 | 1.70 | 0.61 | 1.55 | 0.93 |
| 1:7 | 11.6 | 0.99 | 1.68 | 0.62 | 1.55 | 0.94 |
| - :1 | 12.2 | | | | | |

**Figure S3.** $T_1$ relaxation times (298 K) of H6 proton of 5-FU and selected β-CD signals.

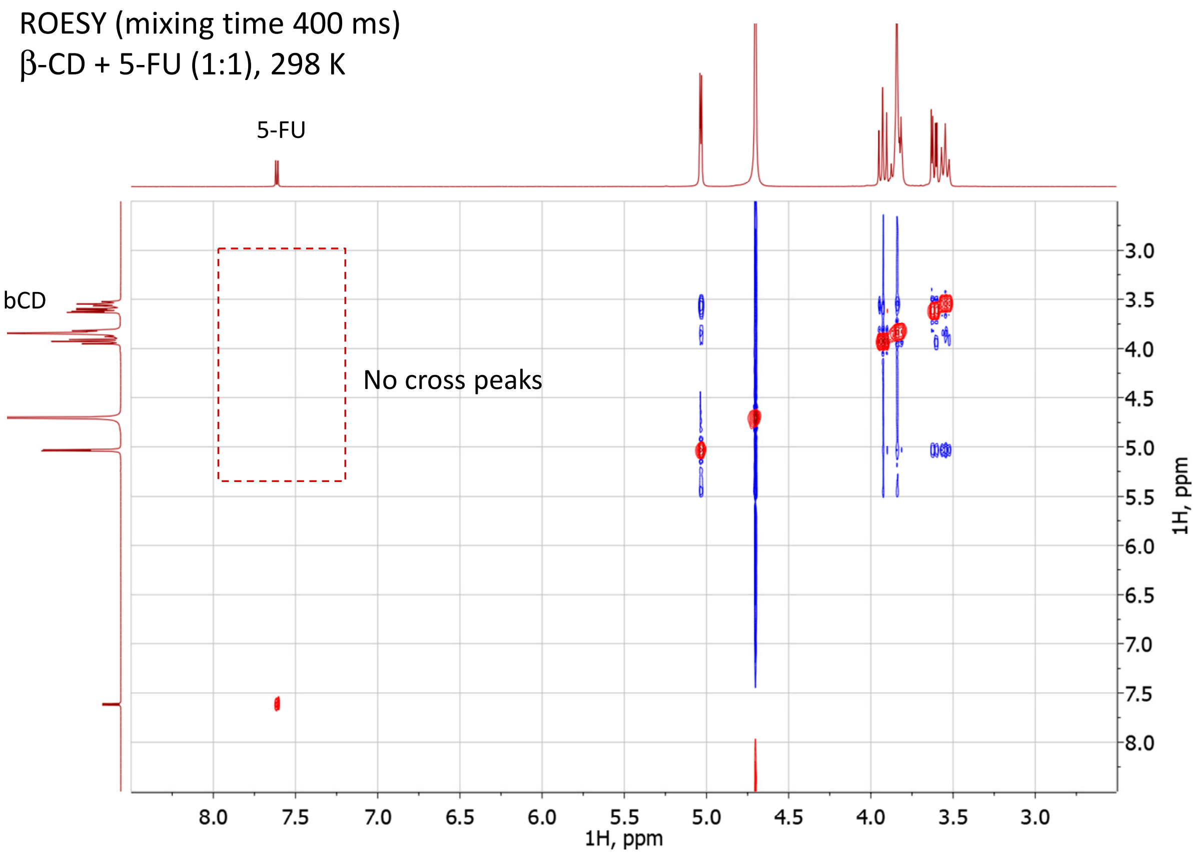


**Figure S4.** ROESY spectrum of 1:1 mixture of 5-FU with β-CD at 298 K. The spectral fragment where cross peaks should appear, in case of complex formation, is marked by a dashed rectangle.
